# The genetically modified human foreskin fibroblast cell line (YhFF#8) stably expressing *Cas9* gene: A lab resource report

**DOI:** 10.18502/ijrm.v22i1.15243

**Published:** 2024-02-23

**Authors:** Farzad Soheilipour, Sohrab Boozarpour, Shiva Aghaei, Ehsan Farashahi Yazd

**Affiliations:** ^1^Stem Cell Biology Research Center, Yazd Reproductive Sciences Institute, Shahid Sadoughi University of Medical Sciences, Yazd, Iran.; ^2^Department of Biology, Faculty of Basic Sciences, Gonbad Kavous University, Gonbad Kavous, Iran.

**Keywords:** Fibroblasts, Cell line, Genetic transduction, CRISPR-Cas9.

## Abstract

**Background:**

Stable Cas9 (CRISPR-associated protein 9)-expressing cell lines have emerged as valuable tools in genetic research, enhancing the efficiency of the CRISPR/Cas9 system and streamlining gene editing procedures. These cell lines enable simultaneous editing of multiple genes and reduce the overall editing time.

**Objective:**

This study aimed to develop a stable human fibroblast cell line capable of genetic conversion into a mutant form, serving as a cellular model for a specific genetic disease. The established cell line facilitates investigation of disease mechanisms, testing of potential treatments, and gaining insights into underlying molecular processes.

**Materials and Methods:**

Human embryonic kidney 293LTV cells were used to produce pseudo-virus particles, while Yazd human foreskin fibroblasts batch 8 (YhFF#8) cells were targeted for genetic modification. Transfection of human embryonic kidney 293LTV cells with pCDH-Cas9 plasmid DNA generated pseudo-viral particles. YhFF#8 cells were transduced and selected using antibiotics. Green fluorescent protein (GFP) detection confirmed successful transduction and selection. Relative expression levels of the *Cas9* gene were determined by quantitative polymerase chain reaction.

**Results:**

The study validated the fidelity of the *Cas9* gene cassette sequence and its transcriptional activity. Transduced YhFF#8 cells exhibited green fluorescence, with antibiotic selection resulting in nearly 100% transduced cells. A reporter *GFP* gene enabled real-time monitoring of YhFF#8-Cas9-GFP-PuroR cells using fluorescence microscopy.

**Conclusion:**

YhFF#8-Cas9-GFP-PuroR cells, labeled and susceptible to genomic editing, provide an optimal source for generating induced pluripotent stem cell lines for future biomedical research.

## 1. Introduction 

Eukaryotic cell lines provide a genetically pure population from a primary cell. The produced cells are often used instead of the primary cells in research. These cell lines have many advantages over primary cells and animal models, such as being economical, easy to use, accessible as a stable source of cells, evaluating the reproducibility of results, and fewer ethical concerns. The usage of cell lines has an important role in the development of different biomedical research fields, like the production of vaccines, the testing of medical products, the production of recombinant proteins, the investigation of the biological role of genes, and the production of artificial tissues (1-5). Among them, engineering human foreskin fibroblast cell lines with site-directed mutations is a suitable source to form a cellular model system for different genetic diseases.

The clustered regularly interspaced short palindromic repeats (CRISPR)/CRISPR-associated protein 9 (*Cas9*) gene editing system is the most advanced cell-engineering technology in generating engineered cell lines (6-9).

However, this technology has some challenges, such as the stability and transfer of the functional parts of this system to the target cells. 2 main methods generally perform the transfer of CRISPR-Cas9 compartments to target cells, the common method is the transferring of the *Cas9 *gene and guides RNA sequence within a DNA construct, and the second one is the direct transferring of the own Cas9 protein and guides RNA sequence to target cells.

The length of the *Cas9 *gene sequence (for about 4 Kbp) could have some considerable negative effects, one of them is the decrease of DNA vector transferring efficiency construct in common method, and another one is the low level of cell transfection efficiency in direct transferring method due to the transient presence of the Cas9 protein (24 hr) (10-13). The last limitation is caused by the transient presence of the *Cas9 *gene or protein in the cells.

However, the stable presence of the *Cas9 *gene is a good strategy that can provide better conditions to cope with such challenges. This solution presents the generating of a stable *Cas9*-expressing cell line as an efficient way to successfully edit even multiple genes and the reducer of the gene editing procedure timing that makes these fibroblast cells the best source for generating induced pluripotent stem cell lines with the aim of usage in different fields of the biomedical research (10, 13-18).

According to these considerations, we decided to modify the YhFF#8 cell line genetically with permanent insertion of the *Cas9* gene.

## 2. Materials and Methods

### Cellular sources and eukaryotic cell culture 

The human embryonic kidney 293T cell line and Yazd human foreskin fibroblasts batch 8 (YhFF#8) were gifted from the Iranian Biological Resource Center and Stem Cell Biology Research Center, Yazd, Iran respectively. The cells were cultured in high glucose Dulbecco's Modified Eagle's Medium media with 10% fetal bovine serum (Gibco, USA).

### Recombinant plasmid construction

The plasmid vectors used included pX330-U6-Chimeric_BB-CBh-hSp Cas9 (a gift from Feng Zhang Addgene, plasmid # 42230; http://n2t.net/addgene:42230; RRID: Addgene_42230), pCDH (Bioscience, USA), pMD2 and psPAX2 (Didier Trono provided the former, Addgene plasmid # 12259; http://n2t.net/addgene:12259 and Addgene plasmid # 12260; http://n2t.net/addgene:12260).

The *Cas9 *sequence of PX-330 was amplified using CCas9 primers pair (Table I) in the polymerase chain reaction (PCR) procedure. The PCR reaction mixture (12.5 µl master mix, 1 µl forward primer, 1 µl reverse primer, 3 µl template, and 7.5 µl dH2O) was prepared and used. The PCR reaction program consists of a pre-denaturation step (94 C for 5 min) and a cyclic stage with 40 repeats. Every cycle included 3 steps: a denaturation step at 94 C for 30 sec, an annealing step at 61.8 C for 30 sec, and the third was an extension at 72 C for 240 sec.

The PCR products after the restriction enzymes digestion procedure with *XbaI* and *EcoRI* (Fermentas, Lithuania) were used in the ligation procedure (ligase enzyme from Fermentas, Lithuania) with the products of restriction enzyme digested pCDH (lentiviral transfer vector). The ligation products transformed the STBL4 strain of *E.coli* (IBRC, Iran). After passing of antibiotic selection, the recombinant plasmid DNA was extracted from selected colonies by kit (Gene Transfer Pioneers, Iran), and these extracted plasmids were sequenced using Sanger's method (ABI 3500) with both CCas9 and QCas9 forward primers. The online BLAST software was used for DNA sequence analysis of the resulting *Cas9* sequences.

### Generation of Cas9-pseudo-lentiviral particles

**Figure 1:**
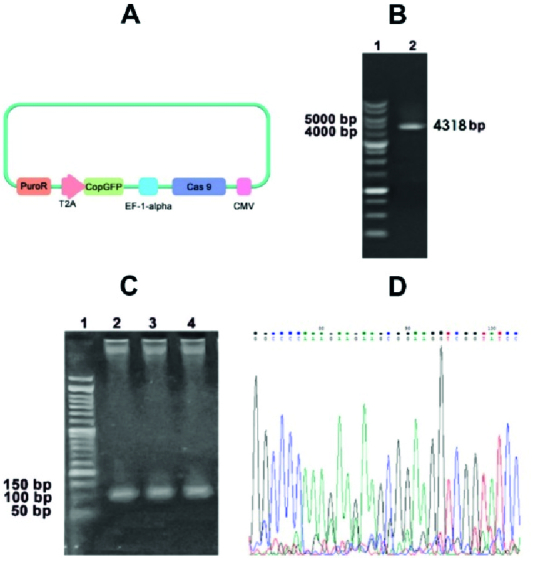
The molecular confirmation of the DNA cloning procedure of the *Cas9* gene. A) A schematic structure of the recombinant pCDH-Cas9 transfer vector. B) The result of the *Cas9* gene fragment amplification (4318 bps). C) The transcription of the 3 plasmids representative genes by RT-PCR in the pseudo-viral particle compartment. Lane 1: *gag* gene (PAX-2 plasmid), Lane 2: *wpre* gene (PMD2 plasmid), Lane 3: *Cas9* gene (pCDH-Cas9 plasmid). D) The DNA sequencing result of the inserted *Cas9* gene of the recombinant pCDH-Cas9 transfer vector.

HEK293LTV cells as the pseudo-viral-producing cell hosts were transfected by the recombinant pCDH-Cas9 transfer vectors (Figure 1A) along with the packaging vectors (pMD2 and psPAX2) to produce pseudo-viral particles via calcium phosphate transfection kit (Bon Yakhteh, Iran). The harvested conditioned media of these cells (48 and 72 hr), which contain produced pseudo-viral particles, were centrifuged and passed through a 0.45 μm filter (Jet Biofil, China) to eliminate dead cells and cellular organelles.

### YhFF#8 cells transduction and antibiotic selection 

The polybrene solution (Sigma Aldrich) was mixed with the filtered harvested media with a final concentration of 8 µg/ml, and it was replaced with the medium of the target cells (YhFF#8). This process was performed 4 times at 12-hr intervals. The antibiotic selection process was carried out using puromycin (Biosun, China) antibiotic with an optimal concentration of 4 μg/ml. The success of this transduction and antibiotic selection procedures was proved via GFP detection under a fluorescence microscope (Olympus, Japan).

### DNA, RNA extraction

Next, the DNA of selected recombinant YhFF#8 cells was extracted based on the DNA extraction kit guidelines (ROJE Technologies, Iran), and its purity and homogeneity were evaluated utilizing the UV spectrophotometer (Thermo Fisher, USA). The total RNA was also extracted from these cells through Riboex solution extraction guidelines (GeneAll, Korea). The purity and the homogeneity of the extracted DNA and RNA were evaluated utilizing a UV spectrophotometer (Thermo Fisher, USA).

### cDNA synthesis and quantitative PCR (qPCR)

The DNase I enzyme (Thermo Fisher, USA) treatment was used to eliminate any DNA remnant in the final extracted RNA. Then cDNA synthesis was done with a reverse transcription kit (Yekta Tajhiz Company, Iran). The relative expression levels of the *Cas9 *gene in comparison with *GAPDH* and *GFP* genes were determined (Table I). The qPCR mixture contained 1 µl of a forward primer, 1 µl of a reverse primer, 10 µl of a SYBR Green Master Mix (Yekta Tajhiz Azma Company, Iran), 2 µl of the sample, and 6 µl of ddH ${\;}_{2}$ O. The PCR reaction program consists of a pre-denaturation step (95 C for 3 min) and a cyclic stage with 40 repeats. Every cycle included 3 steps: a denaturation step at 95 C for 20 sec, the second step, an annealing step at 64 C for 20 sec, and the third was an extension at 72 C for 20 sec. Finally, the results were analyzed with the Excel software and the Pfaffl method (19).

**Table 1:** The sequences of primer pairs


	**Sequence 5 ' → 3 ' **		
**Primer names**	**Forward**	**Reverse**	**Product size**
	**CCas9**	ATATATATATTCTAGAGCCACCATGGACTA		CTGATCAGCGAGCTCTAGGAATTC	4318 bp
**QCas9**	TGAAATACGTGACCGAGGGAATGAG	GTCACTTTCCGGTTGGTCTTGAAC	100 bp
**GAPDH**	CAAGAGCACAAGAGGAAGAGAGAG	TCTACATGGCAACTGTGAGGAG	103 bp
**GFP**	CTTCAGCTACCGCTACGAGG	CGTTGCTGCGGATGATCTTG	104 bp
**WPRE**	GTCCTTTCCATGGCTGCTC	CCGAAGGGACGTAGCAGA	47 bp
**GAG**	AGCGGGGGAGAATTAGATCG	CTGCGAATCGTTCTAGCTCC	112 bp

### Ethical considerations

The current experimental research was conducted by the ethical guidelines established by the Research Ethics Committee of Shahid Sadoughi University of Medical Sciences, Yazd, Iran (Code: IR.SSU.REC.1400.139).

## 3. Results

### The fidelity of the *Cas-9* gene cassette sequence and transcription performance were confirmed

**Figure 2:**
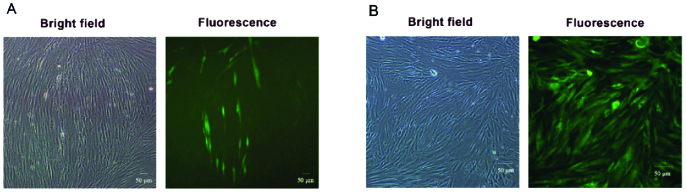
The cellular confirmation of *Cas9* gene transduction to YhFF#8 cells. A) The green fluorescent glowing YhFF#8 cells after transduction and before antibiotic selection. B) The green fluorescent glowing YhFF#8 cells after antibiotic selection.

The integrated *Cas9 *gene into the pCDH vector has 4318 base pairs length (Figure 1B). The upstream sequence of the integration site showed the perfect match with the *Cas9* sequence on the PX330 vector as its DNA template (Figure 1C). The pseudo-viral particle precursors that should be produced in HEK-293T after co-transfection of PMD2, PAX-2, and pCDH-Cas9 plasmids were also confirmed via RT-PCR test (Figure 1D). The transcription of VSV-G, Gag, and *Cas9 *genes in the transfected human embryonic kidney 293T cells was examined to ensure the pseudo-viral particle compartment production. The transduced cells via pseudo-viral particles with the target gene cassette had a green fluorescent emission. The semi-quantitative analysis of microscopic images of these cells showed that 70% of cells emitted green fluorescent light (Figure 2A).

### The YhFF#8-Cas9-GFP cells enrichment by puromycin selection

**Figure 3:**
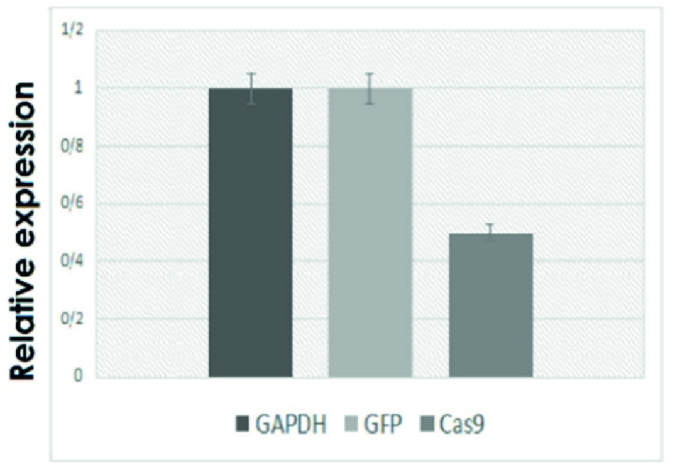
The relative transcription (qPCR result) of the *Cas9* gene compared to the transcription of *GAPDH* and *GFP* genes (as normalizer genes).

The antibiotic selection eliminated the non-transduced cells, and after this process, the percentage of the green-transduced YhFF#8 cells rose in the culture medium to approximately 100% (Figure 2B). Moreover, the relative expression results of these cells by the specific primers of the *Cas9* and *GFP* and, the *GAPDH* internal control gene showed 50% for Cas9 and 100% for GFP transcription (Figure 3).

## 4. Discussion

With the increasing uses of the CRISPR/Cas9 system in different fields of molecular biology research and biotechnology fields, it is reasonable and important that the speed and efficiency of this system will be enhanced. There are certain challenges against the use of this technology including the low transfer rate and the rational low functional stability of its components to target cells (17, 20). The generation of permanent *Cas9*-expressing cell lines is one way of coping with these challenges. This strategy eliminates the simultaneous need for several gene vectors and shortens the duration of genome editing. Moreover, it allows increasing the number of target genes to edit (21, 22).

In such cell lines, it is only necessary to transfer one or several guide RNAs to knock out the genes. The advantages of stability and the maintenance of the Cas9 protein turn the stable *Cas9*-generating cell lines into a confident strategy for genome editing (10, 22, 23). The most important prerequisite for the generation of an engineered human fibroblast cell line is a line of well-characterized genuine fibroblast cells. A major source of human basic fibroblasts with minimal moral concern is the HFFs, which can be obtained from babies or adults who undergo circumcision surgery. The present research made use of a fibroblast cell line taken from young YhFF#8 and already confirmed and developed by Hajizadeh-Tafti et al. (24). Thus, sampling challenges, moral concerns, and such tasks as genuineness confirmation and development were skipped. An important aspect of the cell line generated in this study is the procedure for transducing the *Cas9 *gene using a pseudo-lentivirus vector. The pseudo-virus vectors were created using an engineered pCDH-Cas9 vector, which integrated into the genome of the target cell, resulting in a sustainable genetic change (16, 25). The newly produced cells were then selected and purified using an antibiotic, which was the most effective and cost-efficient method due to the presence of the *PuroR* gene in the pCDH-Cas9 structure.

Another noteworthy point concerns the use of the reporter *GFP* gene in this study, which allowed for real-time monitoring of the transduced cells using a fluorescence microscope. This enabled continuous monitoring of the targeted gene compartment in living cells.

The pCDH-Cas9 vector transfers an expression cassette with 2 independent promoters, cytomegalovirus (CMV) for the *Cas9 *gene and EF-1-α for *GFP* reporter genes and the puromycin resistance gene (shown in Figure 1A). CMV is a promoter derived from cytomegalovirus, while EF-1-α is a eukaryotic and human promoter (26, 27). Although measures have been taken to ensure the proper function of these promoters, some reports have indicated the silencing of genes transferred by pseudo-virus vectors after a certain number of cell divisions (starting from the 4 ${\;}^{\text{th}}$ wk of transduction) (28-30). To assess gene stability, cells were given several more divisions, and subjected to freezing and thawing, and this process was repeated once. The expression of the GFP protein was then confirmed using fluorescence microscope imaging.

Due to intrinsic functional differences between the CMV promoter (related to the *Cas9 *gene) and EF-1-α (related to the *GFP* gene), as well as occasional differences in cells, it was necessary to examine the comparative expression of the *Cas9 *gene. This examination was performed using the qRT-PCR method implemented on selected YhFF#8-Cas9-GFP-PuroR cells, and the results showed that *Cas9* was expressed at 50% of the level of *GFP* and the *GAPDH* housekeeping gene. The performance values obtained in this study are consistent with those of other studies in which the EF-1-α promoter outperformed the CMV promoter by 40-60% (27, 31).

## 5. Conclusion

In summary, the cell line achieved in this study is suitable for basic laboratory applications. However, to detect the presence of proteins or confirm the enzymatic function of Cas9, tests using the Western blot technique and standard knockout reactions for the *GFP* gene inserted into the genome of the presented cell line need to be conducted.

##  Data availability

The data that support the findings of this study are available upon reasonable request from the corresponding author. Due to privacy and ethical considerations, some restrictions may apply to the availability of certain sensitive or confidential data. Requests for data access should be directed to Dr Ehsan Farashahi Yazd (ehsanfarashahi@ssu.ac.ir).

##  Author contributions

All authors had full access to all of the data in the study and took responsibility for the integrity of the data and the accuracy of the data analysis. The concept and design: Ehsan Farashahi-Yazd. The experimental test (acquisition), analysis, or interpretation of data: Farzad Soheilipour, Shiva Aghaei and Sohrab Bouzarpour. Drafting of the manuscript: Farzad Soheilipour, Shiva Aghaei and Ehsan Farashahi Yazd. The critical revision of the manuscript for important intellectual content: Sohrab Bouzarpour, Shiva Aghaei, Ehsan Farashahi Yazd. Statistical analysis: Ehsan Farashahi Yazd. Supervision: Ehsan Farashahi Yazd.

##  Conflict of Interest

The authors declare that there is no conflict of interest.
